# Spatial Patterns of Species Diversity and Phylogenetic Structure of Plant Communities in the Tianshan Mountains, Arid Central Asia

**DOI:** 10.3389/fpls.2017.02134

**Published:** 2017-12-13

**Authors:** Hong-Xiang Zhang, Ming-Li Zhang

**Affiliations:** ^1^Key Laboratory of Biogeography and Bioresource in Arid Land, Xinjiang Institute of Ecology and Geography, Chinese Academy of Sciences, Urumqi, China; ^2^Institute of Botany, Chinese Academy of Sciences, Beijing, China

**Keywords:** community phylogeny, environmental gradients, glacial refugia, phylogenetic diversity, phylogenetic community structure, the Tianshan Mountains

## Abstract

The Tianshan Mountains, located in arid Central Asia, have a humid climate and are biodiversity hotspots. Here, we aimed to clarify whether the pattern of species diversity and the phylogenetic structure of plant communities is affected by environmental variables and glacial refugia. In this study, plant community assemblies of 17 research sites with a total of 35 sample plots were investigated at the grassland/woodland boundaries on the Tianshan Mountains. Community phylogeny of these plant communities was constructed based on two plant DNA barcode regions. The indices of phylogenetic diversity and phylogenetic community structure were calculated for these sample plots. We first estimated the correlation coefficients between species richness (SR) and environmental variables as well as the presence of glacial refugia. We then mapped the significant values of indices of community phylogeny (PD, RPD, NRI, and NTI) to investigate the correlation between community phylogeny and environmental structure or macrozones in the study area. The results showed that a significantly higher value of SR was obtained for the refugial groups than for the colonizing groups (*P* < 0.05); presence of refugia and environmental variables were highly correlated to the pattern of variation in SR. Indices of community phylogeny were not significantly different between refugial and colonizing regions. Comparison with the humid western part showed that plant communities in the arid eastern part of the Tianshan Mountains tended to display more significant phylogenetic overdispersion. The variation tendency of the PhyloSor index showed that the increase in macro-geographical and environmental distance did not influence obvious phylogenetic dissimilarities between different sample plots. In conclusion, glacial refugia and environmental factors profoundly influenced the pattern of SR, but community phylogenetic structure was not affected by glacial refugia among different plant communities on the Tianshan Mountains. This pattern of community phylogenetic structure could have resulted from shared ancestry and species pool among these sample plots.

## Introduction

How species assemble in communities is a fundamental question in ecology and biogeography. Patterns of community assembly usually vary along temporal and spatial gradients (Freestone and Inouye, 2015), but underlying mechanisms of this variation are not clarified yet. Currently, a growing number of ecological and evolutionary hypotheses attempt to address this question. Community phylogenetics, which incorporates phylogenetic information into community ecology, provides an evolutionary framework for investigating community assembly (Webb et al., 2002). Based on the hypotheses on phylogenetic niche conservatism (Crisp and Cook, 2012), species with close phylogenetic relationships should have more similar ecological requirements than distantly related species. Habitat filtering and biotic interactions influence community phylogenies, while biogeographical and ecological processes influence the patterns of community assembly associated with temporal and spatial changes of the environment.

To test these hypotheses, influences of environmental gradients and temporal changes in patterns of community assembly have attracted increasing attention in previous studies. In particular, these studies have focused on the change in community structure along elevation gradients on the mountains. For example, Machac et al. (2011) showed phylogenetic overdispersion in communities at low-elevation sites under interspecific competition, while communities tended to be phylogenetically clustered at high-elevation sites primarily by environmental filtering in temperate montane systems. Qian et al. (2014) also found that plant communities tended to be more phylogenetically clustered at higher elevations in the temperate Changbaishan Mountains. Besides elevation gradients, environmental factors, including edaphic (Fine and Kembel, 2011) and latitudinal gradients (Qian et al., 2013), have been shown to shape the phylogenetic community structure. Several studies have attempted to explore the determinants of the pattern of community assembly from historical and biogeographical processes. Eiserhardt et al. (2015) indicated that spatial patterns in phylogenetic diversity of conifer communities are strongly affected by the late Cenozoic climate history. Because of the influence of the Quaternary climatic changes, phylogenetic evenness was usually detected in refugia with long-term stable habitats, while phylogenetic clustering is more evident in highly disturbed areas after recolonization (Kooyman et al., 2011). These glacial refugia then tend to preserve high phylogenetic diversity (Costion et al., 2015).

Previous studies have focused on various ecosystems throughout the world, but patterns of community assemblage in temperate arid regions have rarely been examined, possibly due to their low level of biodiversity. Central Asia is one of typical arid regions in the temperate zone. In this region, mountain ranges receive more precipitation due to their higher topography, and these mountainous areas are covered by coniferous forests (Hu, 2004). The Tianshan Mountain Range, the largest mountain system in Central Asia, is a biodiversity hotspot and is like an island surrounded by deserts. Aridity of the Asian interior was intensified under the effects of the Qinghai-Tibetan Plateau uplift. The climate of the Tianshan Mountains is dominated by the mid-latitude westerlies, which come from the North Atlantic and Mediterranean and the Siberian High (Xu et al., 2010). Currently, a regional precipitation gradient exists in this mountain range, which decreases from west to east. Besides the variation in precipitation, soil and temperature also change on the Tianshan Mountains from west to east (Hu, 2004).

It was hypothesized that environmental and palaeoclimatic changes had jointly influenced distribution patterns of plant species on the Tianshan Mountains. During the Pleistocene, multiple cycles of cold-dry glacial and warm-humid interglacial climates prevailed in this region (Xu et al., 2010). The western Tianshan Mountains, around the Ili Valley, receive greater amounts of precipitation and serve as a refugium for plant species (Zhang and Zhang, 2012). A high level of plant species diversity is displayed in glacial refugia and humid areas (Zhang and Zhang, 2014). These refugia also preserved high levels of intraspecific genetic diversity for many plant species on the Tianshan Mountains (Zhang and Zhang, 2012; Zhang et al., 2013, 2017). However, the impacts of environmental and biogeographical processes on plant community assembly in these arid mountains have not been studied to date. Thus, it is important to understand the mechanisms of community assembly and the maintenance of biodiversity in temperate arid regions.

In the present study, we investigated species composition of plant communities and built a community phylogeny for the plants inhabiting grassland/woodland boundaries on the Tianshan Mountains. Due to the changes in temperature and precipitation with increasing altitude, different vegetation zones occur in Central Asian arid mountain ranges (Hu, 2004). Among these vertical vegetation zones, the highest level of plant species diversity was shown at grassland/woodland boundaries on the Tianshan Mountains. Meanwhile, plants at the boundaries are controlled by complex environmental and climatic factors, and are particularly sensitive to climate changes (Camill and Clark, 2000; Clark et al., 2001; Ohara and Ushimaru, 2015). The objectives of this study were to: (1) determine the effects of environmental variables and palaeoclimatic changes on spatial patterns of species richness (SR) in plant communities; (2) clarify the pattern of phylogenetic community structure in these plant communities; (3) understand plant community assembly mechanisms on the Tianshan Mountains.

## Materials and methods

### Survey plots and species identification

**Figure 1:**
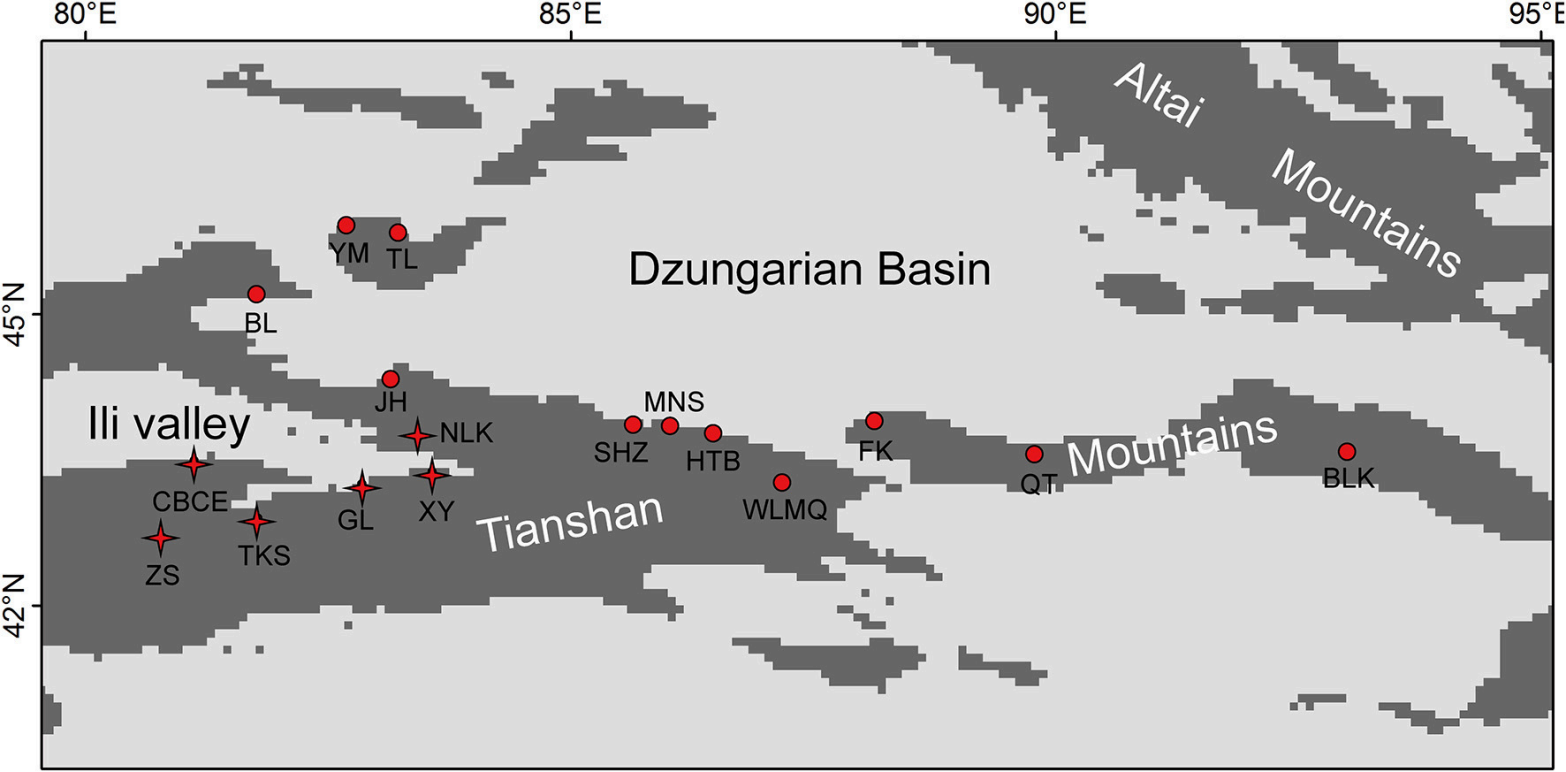
Distribution map of the 17 research sites on the Tianshan Mountains. The sites with “stars” were included in the refugial group, while the sites with “dots” were included in colonizing group.

The Tianshan Mountains extend from west to east and are surrounded by two large arid basins (Figure 1) in eastern Central Asia. Environmental conditions change from west to east, in which the western mountains are more humid than the eastern (annual precipitation: 400 vs. 150 mm, Hu, 2004). Because of its topography, the Tianshan Mountains receive more precipitation than surrounding areas. These mountains are covered by forests of *Picea schrenkiana*, whose flora is affiliated with the Eurasian forest. In the western part of this mountain range, broad-leaved forests occur in patches below the zone of coniferous forest. The altitude of the lower timberline in the eastern part of the Tianshan Mountains is ca. 1,700 m, while it is ca. 1,200 m in the western part of the mountains. Across this mountain range, 17 research sites were sampled with a total of 35 plots (Figure 1, Table 1). Sample plots (1 × 1 m) were located in the grasslands near the lower timberline. Plots were randomly sampled in locations with homogeneous vegetation and away from the influence of anthropogenic disturbances. Based on the standards for vegetation surveys in other studies, 1 × 1 m plots were used to investigate species composition in herb communities (Jiang et al., 2017; Wang et al., 2017). High levels of SR and homogeneous plant species distributions also satisfied the representation of the investigated vegetation using 1 × 1 m plots in the grassland near the lower timberline of the Tianshan Mountains.

**Table 1:** Species richness (SR), phylogenetic diversity (PD), relative phylogenetic diversity (RPD), net relatedness index (NRI), and nearest taxon index (NTI) for 35 plots from 17 research sites on the Tianshan Mountains.

**Plot name**	**SR**	**PD**	**RPD**	**NRI**	**NTI**
BLK-1	14	**0.1475**	2.0631	−1.1532	−0.1697
QT-1	15	0.1582	1.6598	−1.2239	−**2.1374**
QT-2	12	0.1512	1.8133	−1.3013	1.1080
FK-1	12	0.1385	1.6605	−**2.3838**	0.5205
FK-2	10	0.1349	1.8867	−0.4110	0.0948
FK-3	15	0.1617	1.5779	−0.2466	−1.8519
WLMQ-1	17	**0.1729**	1.4511	−1.4490	−0.4374
WLMQ-2	15	0.1664	1.6239	−0.6715	−**2.0574**
WLMQ-3	20	0.1739	1.4033	−0.8204	−0.9642
HTB-1	13	0.1741	1.3789	0.9930	−0.0286
HTB-2	19	0.1589	**1.5883**	−0.1015	−0.5493
HTB-3	18	0.1434	1.7704	0.7109	0.1061
MNS-1	15	0.1399	1.7793	−0.4440	−1.6448
MNS-2	20	**0.1235**	**1.9930**	0.1306	−0.6166
MNS-3	20	0.1415	**1.8002**	−0.9503	−0.3559
SHZ-1	16	0.1357	**1.8371**	−0.2294	−1.8101
SHZ-2	19	**0.1231**	**2.2466**	−0.0004	−0.5385
SHZ-3	14	0.1291	1.8684	−0.5415	−3.0565
JH-1	17	0.1342	1.6567	−0.3743	−1.1535
BL-1	17	0.1392	1.7178	1.0860	0.4079
BL-2	15	0.1347	1.570	1.5585	−0.3073
TL-1	19	0.1415	1.5231	0.1369	0.9495
YM-1	19	0.1379	1.5643	0.5058	1.0113
YM-2	16	**0.1082**	1.6827	1.9020	−0.5231
CBCE-1	11	0.1403	1.8989	−0.9553	−0.6859
TKS-1	19	0.1520	1.6792	0.4271	−0.4355
TKS-2	16	0.1408	1.5546	−1.7076	−1.8259
NLK-1	17	0.1394	**1.8286**	−0.9018	1.3315
NLK-2	17	**0.1386**	1.6159	0.0499	−0.7032
GL-1	25	0.1573	1.4674	−0.6591	−1.4401
GL-2	26	0.1669	1.4590	−0.3412	−0.4477
XY-1	22	0.1639	1.4041	−1.5927	0.2293
XY-2	26	0.1736	1.3249	−0.5089	0.6486
ZS-1	28	0.1736	1.3489	−0.8273	0.0163
ZS-2	26	0.1674	1.5277	−0.7660	−1.1037

*Significant values of these indices are in bold. The 17 research sites are shown in Figure 1*.

All angiosperm species were tagged and identified to assess SR and diversity for each plot. Following preliminary identification in the field, plant species were verified in the laboratory. In total, 92 species were sampled from all plots, representing 81 genera from 32 families. Leaves or entire plants were collected and dried in silica gel for subsequent DNA extraction.

### DNA extraction, amplification, and sequencing

Total genomic DNA was extracted from approximately 50 mg of silica-dried sample leaves or plants following a CTAB protocol modified from Doyle and Doyle (1987). Two DNA regions were sequenced for the plants: a portion of *mat*K and subunit “a” of *rbc*L (Yessoufou et al., 2013). These DNA regions have been recommended as suitable “DNA barcodes” to identify and reconstruct the phylogeny of land plants (CBOL Plant Working Group, 2009). The PCR mixture and amplification program followed the protocols of Zhang and Zhang (2012). PCR products were purified from an agarose gel using the PCR product purification kit following the recommended protocol (Sangon Biotech Co. Ltd., Shanghai, China). Sequencing reactions were performed with the primers described above in both directions by standard methods on an ABI 3730 automated sequencer in Sangon Biotech Co. Ltd., Shanghai, China. Sequences were aligned using CLUSTAL_X (Thompson et al., 1997) and then checked manually. All sequences for these sampling plants were deposited in GenBank Databases under accession numbers: MF158641-MF158810.

### Phylogenetic analysis

We estimated phylogenetic relationships among the 92 plant species that occurred in these sample plots. To construct the topology of this phylogenetic tree, the construction method of Kress et al. (2010) was employed with application of constraint trees. This approach defined the backbone of the tree at the family level in accordance with APG III (Bremer et al., 2009), but within each family, the positions of species were resolved with the barcode sequence data. According to this method, a phylogenetic tree was first generated for the sampled species using the online program Phylomatic (Webb and Donoghue, 2005). This tree specified the taxonomic relationship of the 32 families, but within each family species were arrayed as a polytomy. The topology of this tree was defined as the constraint tree in the following phylogenetic construction. A DNA supermatrix of two markers (*mat*K + *rbc*L) was then generated. A maximum likelihood (ML) community phylogeny was performed using RAxML (Stamatakis, 2006) via the CIPRES supercomputer cluster (Miller et al., 2010) based on the DNA supermatrix and the constraint tree. Node support was estimated using bootstrap values with 500 replicates. Finally, we calibrated the branch lengths of this ML phylogenetic tree using the program r8s v1.70 (Sanderson, 2003) with the NPRS method, which was employed in the analysis of the phylogenetic structure of plant communities. We used seven fossil calibration points. Minimum ages were set at the crown node of Rosales (70 Ma), Fabales (70 Ma), Caryophyllales (80 Ma), Lamiales (70 Ma), Asterales (80 Ma), Ranunculales (110 Ma), and Poales (80 Ma) (http://www.mobot.org/MOBOT/research/APweb/; Bremer et al., 2009).

### Community phylogenetic diversity and structure analyses

Plant community diversity in each plot was quantified using three different metrics. SR is simply a count of the number of terminal taxa in each sample plot; the phylogenetic diversity (PD) in each sample plot, which is the total length of the branches connecting these terminal taxa (Faith, 1992), was calculated in the program Biodiverse (version 1.0; Laffan et al., 2010); and the Phylobetadiversity PhyloSor index represents the proportion of shared branch lengths between pairs of plots (Bryant et al., 2008), which was calculated in the R-package Picante v1.2 (Kembel et al., 2010). High values of the PhyloSor index indicate that phylogenetic compositions are similar between the corresponding plots.

Phylogenetic community structure was quantified using three indices for these sample plots: the relative phylogenetic diversity (RPD), the net relatedness index (NRI) and the nearest taxon index (NTI) (Webb et al., 2002). The RPD was calculated in the program Biodiverse (version 1.0: Laffan et al., 2010) while the NRI and NTI were implemented in the R-package Picante v1.2 (Kembel et al., 2010). The RPD provides ratios that compare the PD observed on the actual tree in the numerator to that observed on a comparison tree in the denominator. A significantly high RPD shows an area where there is an over-representation of long branches and phylogenetic overdispersion, while a significantly low RPD shows an area where there is an over-representation of short branches and phylogenetic clustering (Mishler et al., 2014). NRI is relative to a phylogeny of an appropriate species pool and quantifies overall clustering of taxa on a tree, whereas NTI is independent of deep level clustering and quantifies the extent of terminal clustering. For both metrics, positive values indicate that species are more closely related than predicted by chance in local assemblages (phylogenetic clustering), whereas negative values indicate that species are more phylogenetically diverse than expected by chance in local assemblages (phylogenetic overdispersion). The NRI and NTI are calculated as follows: NRI = −1 × (MPD − MPD_rand_)/sd(MPD_rand_); NTI = −1 × (MNTD − MNTD_rand_)/sd(MNTD_rand_). MPD represents an overall measure of phylogenetic diversity within a local assemblage, and MNTD quantifies distances between nearest neighbors within a local assemblage. The MPD_rand_ and MNTD_rand_ represent the mean MPD and mean MMPD from 999 randomly generated assemblages. The sd(MPD_rand_) and sd(MNTD_rand_) represent standard deviation of MPD and MMPD under the 999 randomly generated assemblages. These 999 random assemblages were generated using an independent swap null model (Gotelli and Entsminger, 2001).

To test the significance of PD and RPD, a randomization with a null model that retained some of the structural features of the data was used in the program Biodiverse (version 1.0; Laffan et al., 2010). We ran 999 trials of the randomization null model to calculate PD and RPD for each trial. Then, these values formed a null distribution for each plot, and a two-tailed test was applied to examine whether these values were significantly higher or significantly lower than the null model. If the observed value fell into the highest or the lowest 2.5% of the distribution for that plot, it was judged to be significant (*P* = 0.05).

### Statistical analysis

Based on previous studies on species diversity and molecular phylogeography, the mountains around the Ili Valley provided glacial refugia for forest plants on the Tianshan Mountains (Zhang and Zhang, 2012, 2014; Zhang et al., 2017). During glacial periods, a cold-arid climate prevailed on the Tianshan Mountains (Xu et al., 2010). The mid-elevation locations from the mountains around the Ili Valley provided humid and warm refugia to preserve high levels of species and genetic diversity. In this study, we defined these sample plots from the mountains around the Ili Valley (Figure 1) as the refugial group, and other sampling plots were defined as colonizing group.

We initially calculated the difference of SR between the refugial and colonizing groups. Significance was tested by means of the Mann–Whitney *U*-test in SPSS v17.0. Then, the correlation coefficients between SR and 25 environmental variables as well as the presence of glacial refugia were calculated, which aimed to determine the influence of environmental variables and glacial refugia on species diversity of these sampled communities. These 25 environmental variables included altitude, 19 bioclimatic variables (available at the WorldClim database; http://www.worldclim.org/; Hijmans et al., 2005), and five soil variables [available at the International Geosphere-Biosphere Programme Data and Information System (IGBP-DIS) database; Global Soil Data Task Group, 2000]. These analyses were performed using SPSS v17.0.

To display the correlation between community phylogeny and environmental structure or macrozones, we mapped the spatial distribution of significant PD, MPD, NRI, and NTI on the Tianshan Mountains. In addition, we related the PhyloSor index to geographical distance and environmental distance. Geographical distance between plots was measured using geographical coordinates; environmental distance was measured using the Euclidean distance of these 25 environmental variables among pairs of plots.

## Results

In total, 92 plant species were recorded in 35 survey plots from 17 research sites. In each sample plot, SR ranged from 10 to 26 (Table 1). The research sites of XY, GL, and ZS displayed the highest levels of SR, whereas research sites of FK, QT, and CBCE displayed the lowest levels of SR.

**Figure 2:**
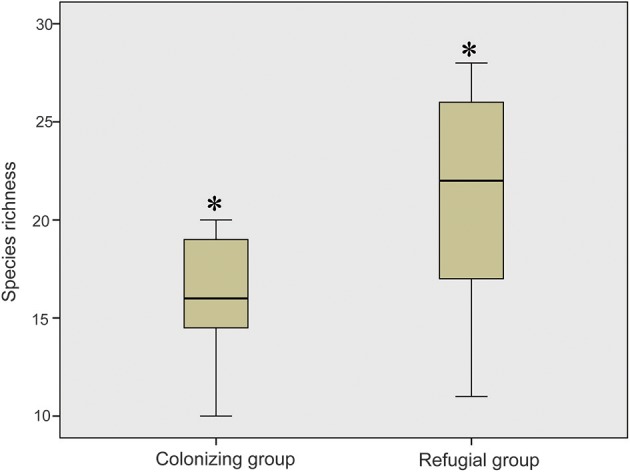
Comparison of the values of the species richness between colonizing group and refugial group (Single asterisk shows significance at the *P* < 0.05 level).

**Figure 3:**
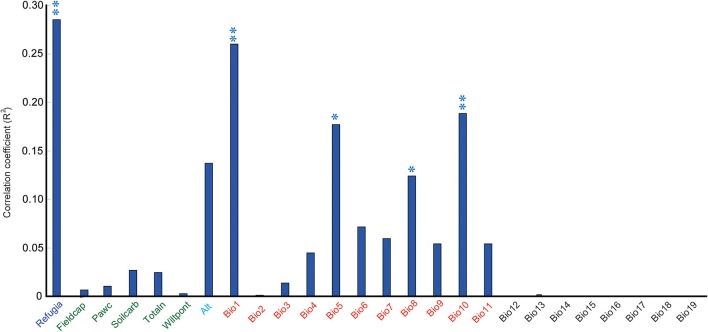
Correlation coefficients between the species richness (SR) and 25 environmental variables and the LGM refugia (double asterisk shows significance at the *P* < 0.01 level; single asterisk shows significance at the *P* < 0.05 level). The detailed information on 25 environmental variables was shown in Supplementary Table 1.

When these research sites were divided into refugial and colonizing groups, significantly higher values of SR were obtained for refugial groups than for colonizing groups (*P* < 0.05; Figure 2). In the correlation analysis, values of SR for these sample plots were significantly related to the location of refugia, altitude, and four temperature variables (Annual Mean Temperature, Max Temperature of Warmest Month, Mean Temperature of Wettest Quarter, and Mean Temperature of Warmest Quarter; Figure 3).

**Figure 4:**
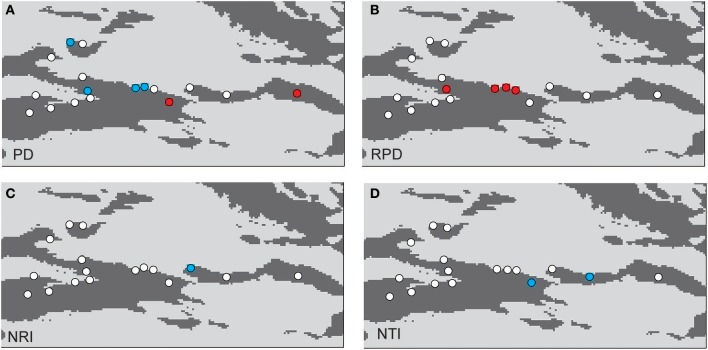
Maps showing significant values resulting from of a randomization test: **(A)** PD, **(B)** RPD, **(C)** NRI, and **(D)** NTI. The red circles indicate plots with significantly higher values than expected; the blue circles indicate plots with significantly lower values than expected; the white circles are not significant.

**Figure 5:**
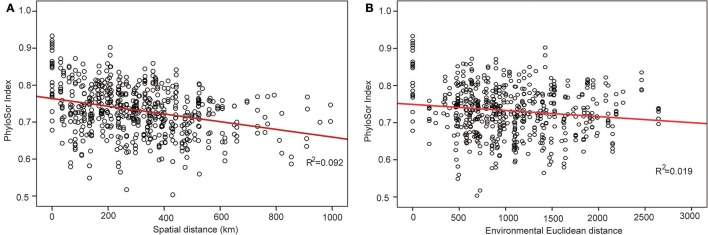
Change in the PhyloSor index following with the increase in **(A)** geographical distance and **(B)** environmental distance.

The phylogenetic tree of the 92 plant species yielded high support values, in which bootstrap values of most nodes were >90% (Supplementary Figure 1) based on the backbone of the APG III constraint tree and terminal DNA barcode resolving. PD values of 35 sample plots were estimated to range from 0.1082 to 0.1741 (Table 1). The significantly high PD values occurred at the research sites of BLK and WLMQ, while the significant low occurred at the research sites of MNS, SHZ, YM, and NLK (Figure 4, Table 1). For the three indices of phylogenetic community structure, the significantly high RPD values occurred at the research sites of HTB, MNS, SHZ, and NLK (Figure 4, Table 1); the significant negative values of NRI and NTI were shown at the research sites of FK, QT, and WLMQ (Figure 4, Table 1). Phylogenetic similarity between different sample plots using the PhyloSor index did not display any obvious correlation with the increase in geographical or environmental distance (Figure 5).

## Discussion

### Impact of glacial refugia and environmental factors on species richness of plant communities

Glacial refugia, such as the mountains around the Ili Valley, displayed high levels of species diversity on the Tianshan Mountains. The hypothesis that glacial refugia preserved high levels of plant species diversity has been tested and supported in some ecosystems, including the Mediterranean Basin (Médail and Diadema, 2009), Amazonia (Hammen and Hooghiemstra, 2000), and subtropical Australia (Weber et al., 2014). In the present study, species composition of sample plots also showed that glacial refugial regions preserved significantly higher SR than colonizing regions (Figure 2) on the Tianshan Mountains of arid Central Asia. The mountains around the Ili Valley were recognized as refugial regions on the Tianshan Mountains (Zhang and Zhang, 2014; Zhang et al., 2017). The climate of this area is humid and might be resistant to the increasing aridity during glacial periods. Therefore, these refugial regions provided stable and suitable harbors to preserve high levels of plant SR. Apart from higher levels of species diversity, refugial regions also held higher levels of genetic diversity compared to colonizing regions within a single plant species on the Tianshan Mountains (Zhang and Zhang, 2012; Zhang et al., 2013, 2017). These refugial regions served as biodiversity hotspots on the Tianshan Mountains from both species and genetic perspectives.

The pattern of species diversity on the Tianshan Mountains was previously revealed to be highly associated with environmental conditions based on species distribution modeling (Zhang and Zhang, 2014). High species diversity always occurred in regions with greater precipitation. In the present study, data collected from sample plots showed that the level of SR was related to altitude and temperature variables (Figure 4), which was different from the results of species distribution modeling. The reason was that plots in this study were sampled from the same vegetation zone at the lower timberline-grassland ecotone, while the precipitation condition was similar in the same vegetation zone along the Tianshan Mountains.

### Roles of glacial refugia and environmental factors on community phylogeny

The regional-scale pattern of community phylogeny can be constructed based on the phylogenetic relationships of all taxa from multiple communities. Effects of biogeographical processes on the community assembly were then elucidated in this region. Historical events, environmental filtering and species competition were proposed as major factors influencing community assembly (Webb et al., 2002). A hypothesis suggested that high evolutionary potential is maintained in refugial regions with characteristics of high phylogenetic diversity, which has been verified in other ecosystems (Tolley et al., 2011; Gavilanez and Stevens, 2013; Costion et al., 2015). During the long-term history of palaeoclimatic changes, plant communities in refugial regions could have accumulated more evolutionary and phylogenetic potential. However, in the present study, most PD values were not significant in plant communities from the Tianshan Mountains (Figure 4, Table 1). Refugial regions did not have significantly higher PD values than colonizing regions (Figure 4). This result indicates that palaeoclimatic changes did not determine the pattern of phylogenetic diversity in plant communities from this arid mountain ecosystem. Based on the spatial distribution of significant PD values (Figure 4), the significantly high PD values occurred in the eastern part of the Tianshan Mountains, while the significantly low PD values were shown in the west. From the basic values of PD (Table 1), we found that western plots had non-significantly high PD values. Western plant commities were expected to have a higher PD than current PD values under the high level of SR. On the Tianshan Mountains, the western part is more humid than the eastern part (Hu, 2004). However, low PD values were likely significant in humid western regions and high PD values were likely significant in arid eastern regions. Within the same research site, such as WLMQ, the PD value of one plot was significant, but other plots were non-significant. These neighbor plots were usually sampled from different microtopographical areas. It seems that the changes in environmental factors could play an important role in shaping the pattern of phylogenetic diversity on the Tianshan Mountains. Similarly, the environmental gradient was considered a primary factor to influence phylogenetic diversity of plant communities in mountain ranges (Dehling et al., 2014; Qian et al., 2014).

The pattern of phylogenetic community structure was hypothesized to be affected by palaeoclimatic changes (Eiserhardt et al., 2015). Phylogenetic evenness is more evident in areas with long-term stability of suitable habitats (e.g., refugia), while phylogenetic clustering is more prevalent in unstable regions (e.g., recolonizing regions) where species pools were more highly disturbed and reduced by historical changes (Kooyman et al., 2011). In the present study, most plots did not show significant values of phylogenetic community structure according to three employed indices (RPD, NRI, and NTI; Table 1). Therefore, the hypothesis that plant communities in glacial refugia were likely to show phylogenetic overdispersion was not supported in the arid Tianshan Mountains. Also, this tendency toward phylogenetic evenness in refugial regions where species interact in conserved niches was not supported by assessing the spatial phylogenetic structure of vertebrate communities across the Australian arid zone (Lanier et al., 2013). In the basic values of phylogenetic community structure (Table 1), RPD values were high, and NRI and NTI values were negative in most sampling plots. From the spatial distribution of significant values of phylogenetic community structure (Figure 4), phylogenetic clustering was not detected in these sampling plots. This showed that the impact of environmental filtering would not have influenced the pattern of phylogenetic community structure. By comparison with the humid west, plant communities in the arid eastern Tianshan Mountains tended to display significant phylogenetic overdispersion (Figure 4).

### Biogeographical and evolutionary mechanisms that influence community phylogeny

Explaining patterns of SR and community phylogeny in montane regions is a critical issue for biodiversity research. To explain these patterns in montane regions, two independent hypotheses have been proposed: the montane species-pump hypothesis and the montane museum hypothesis (Smith et al., 2007; Wiens et al., 2007; Hutter et al., 2013). The montane species-pump hypothesis predicts that biodiversity hotspots have higher speciation rates. The montane museum hypothesis predicts that clades colonized regions of high biodiversity early in their history, leaving more time for species accumulation in these habitats. In the present study, glacial refugia preserved significantly higher levels of SR (Figure 2) than colonizing regions on the Tianshan Mountains. However, these refugial regions did not display significant differences in phylogenetic diversity in comparison to colonizing regions (Figure 4, Table 1). On the Tianshan Mountains, plant species originated much earlier than Quaternary glaciation cycles when intraspecific genetic divergence was promoted for most plants in the study area (Zhang and Zhang, 2012; Zhang et al., 2013, 2017). During glacial periods, refugial regions provided stable and suitable habitats to preserve high levels of plant SR. During the interglacial periods, colonization from refugia to colonizing regions was one of the major driving forces that shaped the structure of community phylogeny rather than *in situ* speciation in this mountain area. The mountain range served as a migration corridor for plant species recolonization. Subsequently, most plant communities were assembled from similar plant lineages across the Tianshan Mountains via migration corridors.

This hypothesis was supported by the variation in the PhyloSor index, where the increase in macro-geographical and environmental distance did not influence obvious phylogenetic dissimilarities between different sample plots (Figure 5). This result indicated that ancestry was shared among these sample plots along with macro-geographical and environmental changes. In the community assembly process after Quaternary glaciations, major plant lineages in the level of orders and families migrated from the refugial regions to the colonizing regions along the corridor of the Tianshan Mountains (Zhang and Zhang, 2014). Although the refugial region served as the montane museum (Hutter et al., 2013) and preserved high levels of SR, major plant lineages migrated to the colonizing regions and did not cause variation in the pattern of community phylogenetic structure across the Tianshan Mountains.

In conclusion, glacial refugia have profoundly influenced the geographical SR pattern among different plant communities on the Tianshan Mountains. Refugial regions provide stable and suitable habitats to preserve a great number of plant species despite palaeoclimatic changes. SR correlated with elevation and temperature changes. In contrast, indices of community phylogeny (PD, RPD, NRI, and NTI) were not significantly different between glacial refugia and colonizing regions. Similarly, phylogenetic dissimilarity between different sample plots was not related to macro-geographical or environmental changes. The results suggest that refugial regions served as montane museums. Major plant lineages in the level of orders and families migrated from the refugial regions to colonizing regions along the corridor of the Tianshan Mountains after Quaternary glaciations. Therefore, ancestry and the species pool were shared among these sample plots. On the Tianshan Mountains, significant differences in SR between refugial and colonizing regions were not reflected in their community phylogenetic structure.

## Author contributions

H-XZ, M-LZ: Conceived and designed the experiments; H-XZ: Performed the experiments; H-XZ: Analyzed the data; H-XZ: Contributed reagents, materials, analysis tools; H-XZ, M-LZ: Wrote the paper.

### Conflict of interest statement

The authors declare that the research was conducted in the absence of any commercial or financial relationships that could be construed as a potential conflict of interest.
