# Supplementary material for: Spatial Patterns of Species Diversity and Phylogenetic Structure of Plant Communities in the Tianshan Mountains, Arid Central Asia
Source: Front Plant Sci. 2017 Dec 13;8:2134. doi: 10.3389/fpls.2017.02134 (PMC5733559; doi:10.3389/fpls.2017.02134)
Supplement: Supplementary file 1 [file Presentation1.PDF]

Supplementary Table 1. The detailed information on twenty-five environmental variables used in the study to analysis their correlation with the index of species richness (SR).

Abbreviations	Full names
Alt	Altitude
Fieldcap	Field Capacity
Pawc	Profile Available Water Capacity
Soilcarb	Soil-Carbon Density
Totaln	Total Nitrogen Density
Wiltpont	Wilting-Point
Bio1	Annual Mean Temperature
Bio2	Mean Diurnal Range
Bio3	Isothermality
Bio4	Temperature Seasonality
Bio5	Max Temperature of Warmest Month
Bio6	Min Temperature of Coldest Month
Bio7	Temperature Annual Range
Bio8	Mean Temperature of Wettest Quarter
Bio9	Mean Temperature of Driest Quarter
Bio10	Mean Temperature of Warmest Quarter
Bio11	Mean Temperature of Coldest Quarter
Bio12	Annual Precipitation
Bio13	Precipitation of Wettest Month
Bio14	Precipitation of Driest Month
Bio15	Precipitation Seasonality
Bio16	Precipitation of Wettest Quarter
Bio17	Precipitation of Driest Quarter
Bio18	Precipitation of Warmest Quarter
Bio19	Precipitation of Coldest Quarter

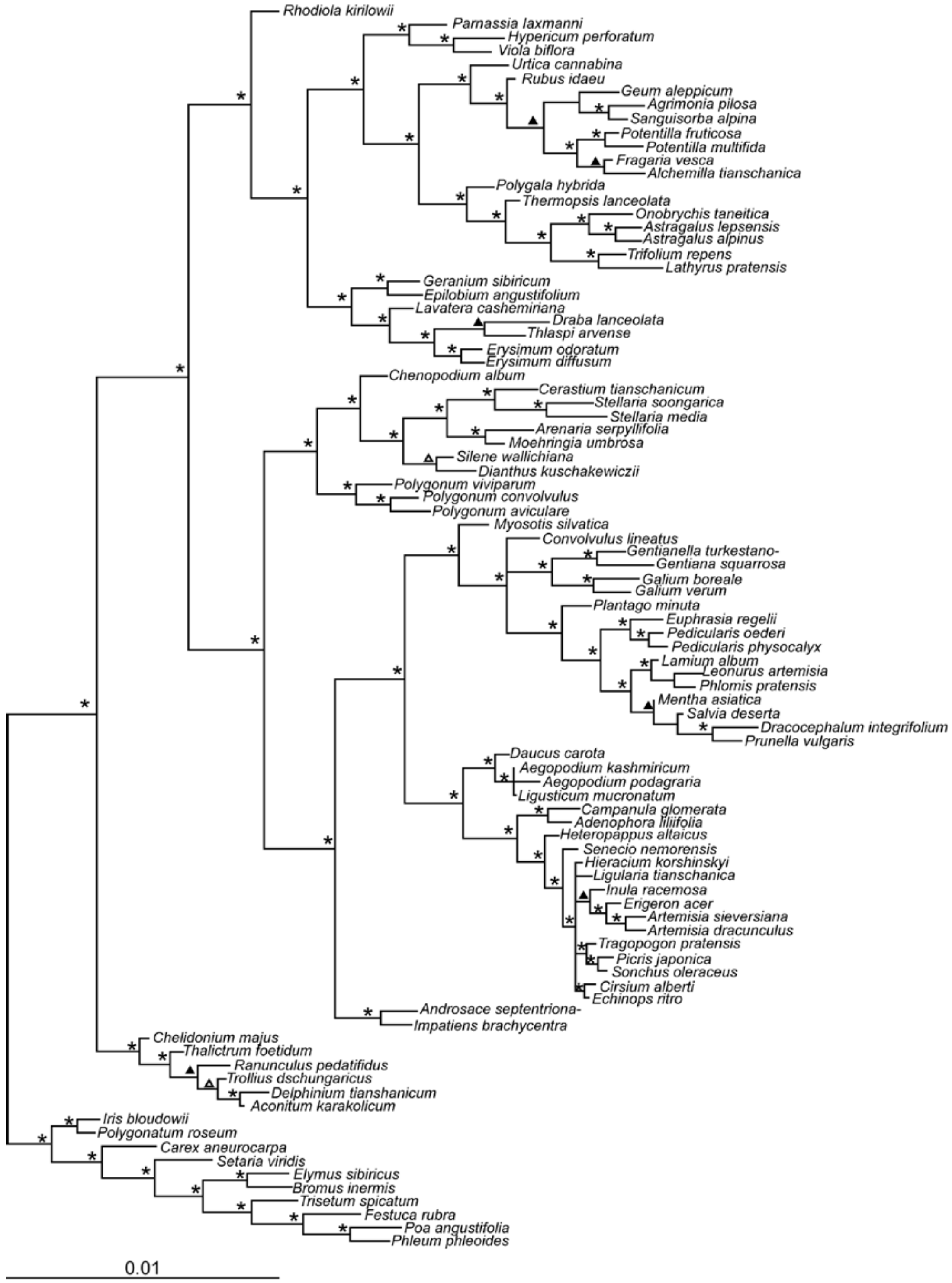

Supplementary Figure 1. Maximum likelihood phylogenetic tree of 92 species of plant community from the Tianshan Mountains based on a supermatrix analysis of *rbc*La and matK sequence data. Nodes with strong support value (>85%) are indicated by an asterisk, nodes with moderate support value (70–85%) are indicated by a solid triangle and nodes with weak support value (50–70%) are indicated by an open triangle.
